# The high-pressure behaviour of caesium hydroxide monohydrate

**DOI:** 10.1039/d6cp03154d

**Published:** 2026-09-24

**Authors:** Keishiro Yamashita, Ciprian G. Pruteanu, Graeme J. Ackland, Kazuki Komatsu, Takanori Hattori, Shinichi Machida, John S. Loveday

**Affiliations:** a SUPA, School of Physics and Astronomy and Centre for Science at Extreme Conditions, University of Edinburgh James Clerk Maxwell Building, Peter Guthrie Tait Road Edinburgh EH9 3FD UK kyamashi@ed.ac.uk cip.pruteanu@ed.ac.uk; b Geochemical Research Center, Graduate School of Science, The University of Tokyo, Bunkyo-ku Tokyo 113-0033 Japan; c J-PARC Center, Japan Atomic Energy Agency 2-4 Shirakata, Tokai, Naka Ibaraki 319-1195 Japan; d Neutron Science and Technology Center, Comprehensive Research Organization for Science and Society (CROSS) IQBRC Building, 162-1 Shirakata, Tokai, Naka Ibaraki 319-1106 Japan

## Abstract

Although the anhydrous alkali hydroxides have been extensively studied, their hydrates have been much less explored despite the common nature of the combination of ionic interactions and hydrogen bonding. In this study, we characterised the compression behaviour of CsOH·H_2_O, which contains a quasi-2D hydrogen-bonded network exhibiting high solid-state proton conductivity *via* the Grotthuss mechanism. No clear structural transition was observed up to 9 GPa, whilst Raman scattering exhibits slight changes in the spectrum at 3 GPa. X-ray and neutron diffraction revealed anisotropic compression of CsOH·H_2_O with the largest compressibility along the *c*-axis, reducing the interlayer distances like the other layered materials. Accordingly, the quasi-2D hydrogen-bonded network becomes flattened. In Raman spectroscopy, a typical pressure response was found for hydrogen-bonded crystals, such as pressure-induced strengthening of hydrogen bonds characterised by the downshift of OH stretching modes as well as the upshift of other optical modes of translations and librational movements. On the other hand, some of the low-frequency bands became indistinguishable above 3 GPa, where the pressure dependence of the OH stretching mode has a kink as well. The anisotropic compression behaviour reduces the free volume, which will affect the peculiar phase behaviour and proton dynamics in the quasi-2D hydrogen-bonded network of CsOH·H_2_O present at ambient pressure.

## Abstract 2

The quasi-2D hydrogen-bonded network of caesium hydroxide monohydrate exhibits anisotropic compression behaviour accompanied by spectroscopic signatures.

## Graphical abstract

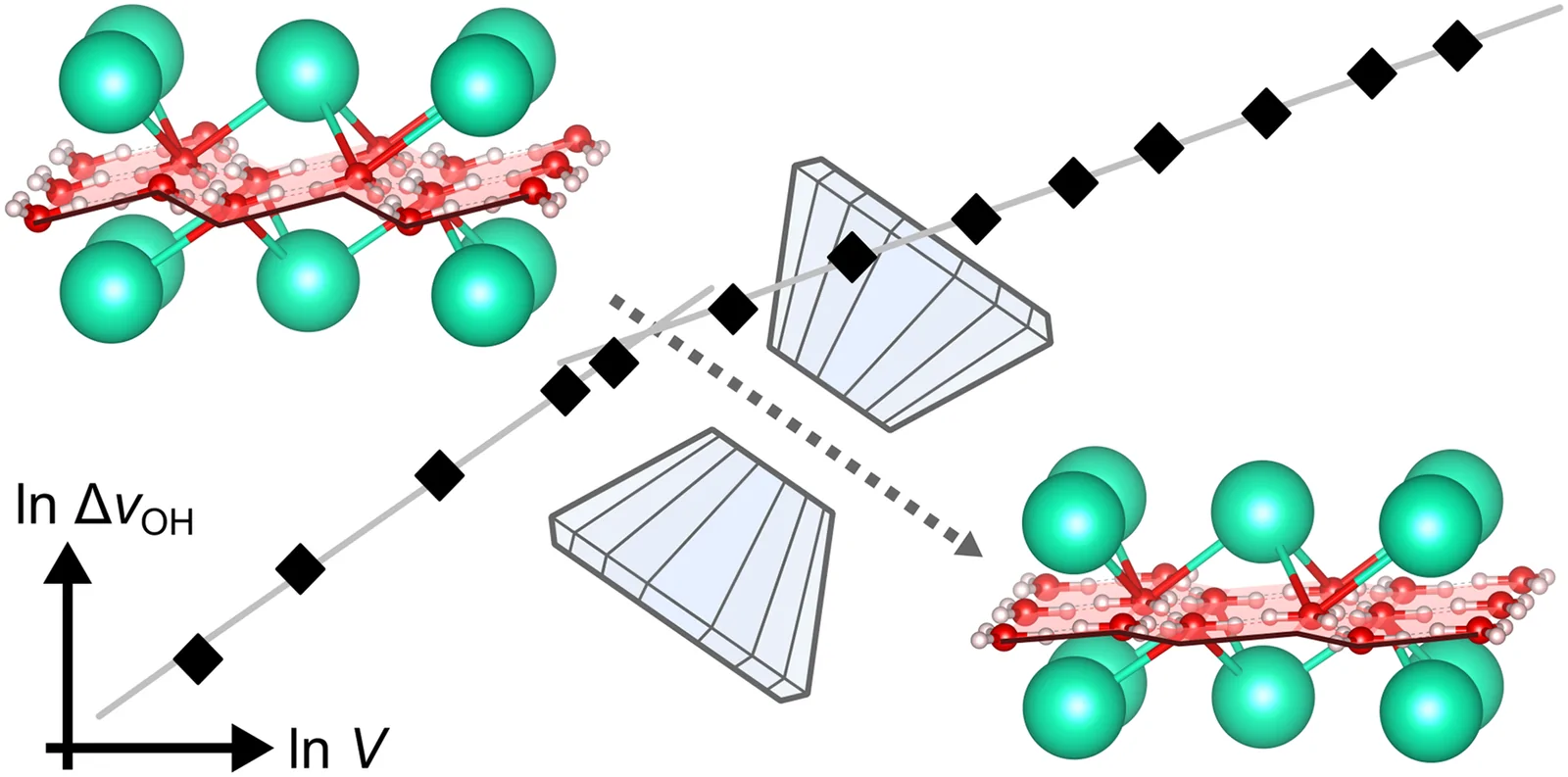

Alkali hydroxides (general formula MOH, where M = Li, Na, K, Rb, Cs) have long been a point of interest in the physics and chemistry community, because they show a fine and easily tunable balance between ionic interactions and hydrogen bonding.^1^ Additionally, alkali hydroxides are also the simple analogues for hydrous minerals, phases which occur in the MgO–SiO_2_–H_2_O and Al_2_O_3_–SiO_2_–H_2_O systems.^2,3^ These phases are crucial for models of the transport and storage of water in the Earth's mantle. At ambient temperatures, the hydrogen bonds in the alkali hydroxides are ordered, resulting in orthorhombic (CsOH^4^ and RbOH^5^), monoclinic (KOH^6^), or tetragonal (LiOH^7^) phases. In contrast, the alkali hydroxides all form a cubic NaCl-type structure at high temperatures,^4,5,8^ with the alkali metals sitting on one lattice site and the OH groups freely rotating.^9,10^ Obviously, in this situation, the structure is governed by ionic bonding between the M^+^ and the OH^−^ group.

Whilst the hydroxides have been the subject of numerous experimental^11,12^ and computational^13^ studies, all showing a very strong pressure effect on the structure and behaviour of the O–H group, little work has been done on the hydrates of these hydroxides. One study dating back more than 30 years^14^ has examined the effect of temperature on the crystal structure of CsOH·H_2_O, but only focused on the system at modest pressures and used X-rays to study the crystal structure (and was therefore unable to locate the H atoms). The phases identified so far in CsOH·H_2_O are as follows: monoclinic (below 232 K, a yet unknown space group), trigonal (232–340 K, *P*3̄*m*1), and a hexagonal phase (above 340 K, *P*6/*mmm*).^14^ All of these phases have a common structural framework with lattice parameters *a* = 4.54 Å and *c* = 4.45 Å at 295 K in the trigonal/hexagonal unit cell.^14^ The trigonal and hexagonal phases exhibit disordered H atoms, which suggests the orientational disorder of molecules. Following the transition behaviour of water-ice phases,^15^ the low-temperature monoclinic phase is expected to be a hydrogen-ordered variant of the higher-temperature structures. Between the trigonal and hexagonal phases, the trigonal phase has a substitutionally ordered H_2_O–OH^−^ arrangement whilst the hexagonal phase exhibits substitutional disorder so that each oxygen site has an equal probability of hosting a water molecule or a hydroxide ion. Such substitutional disorder is not common in such water-based hydrogen-bond network systems.

A diffraction study has looked carefully into the crystal structures adopted by the hydroxide hydrate at various temperatures (and ambient pressure), trying to locate the H-atoms using neutrons.^16^ The authors reported that the space group of the hydrate is more likely to be *P*3*m*1 at temperatures between 232 K and 340 K (in contrast to *P*3̄*m*1 reported by Jacobs *et al.*^14^). The difference between the two models is the (non-)existence of the inversion centre between the two oxygen sites (Fig. 1). These sites are crystallographically equivalent in the *P*3̄*m*1 model and inequivalent in the *P*3*m*1 model. This assessment was based on the *R*_I_ and *R*_wp_ factors of the Rietveld refinements, yielding far more favourable values for the latter structure than the former. It is worth mentioning that the neutron-diffraction measurements were performed at 295, 383 and 414 K, reconfirming the structures proposed initially by Jacobs.^14^ Unfortunately, no data were collected at lower temperatures, so the exact space group for the monoclinic phase still remains unknown.

**Fig. 1:**
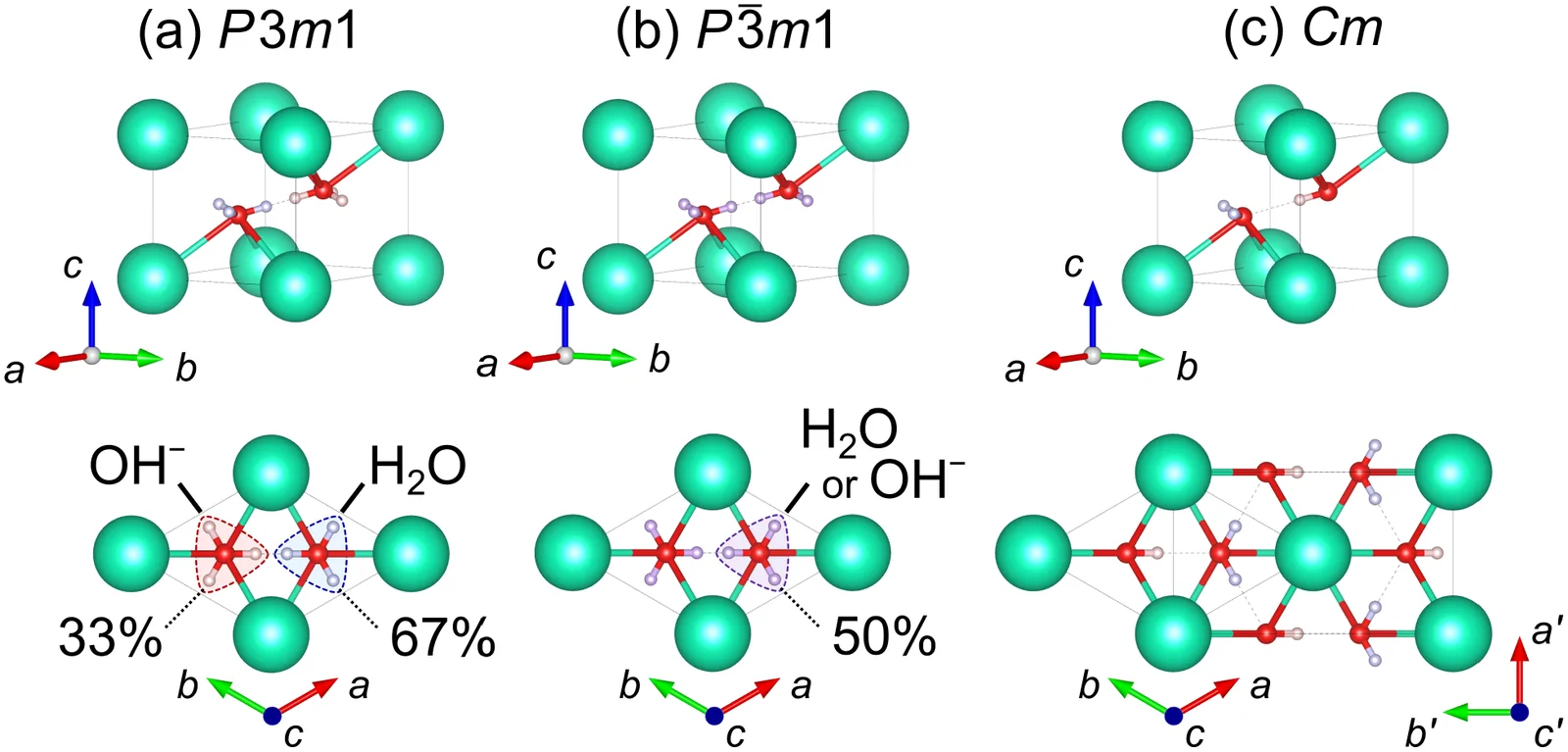
Structure models of CsOH·H_2_O in (a) *P*3*m*1 symmetry adopted in this study, (b) *P*3̄*m*1 symmetry containing positional disorder between H_2_O and OH^−^, and (c) *Cm* symmetry as an ordered configuration used for the DFT calculation. Green and red balls correspond to caesium and oxygen atoms. Small balls correspond to hydrogen, whilst the blue, pink, and violet colours are used to distinguish H_2_O, OH^−^, and their positional disorder, respectively.

Another neutron study performed on CsOH·H_2_O has put forward the idea that in the high-temperature phases the compound exhibits proton diffusion through H jumps between the two sites^17^ and that at 402 K the hydrogens are dynamically disordered. In addition, there has been a suggestion that caesium hydroxide monohydrate has a high-pressure clathrate-type structure,^18^ although this study produced the compound as a by-product in a high-pressure experiment for a different purpose, and determined the crystal structure at ambient pressure for the recovered sample. The authors reported a tetragonal polymorph at 293 K, with space group *I*4_1_/*amd*, but as with most previous studies, they failed to identify the positions of the hydrogen atoms because they used X-rays. Since no information was obtained on H positions, the question of whether they are ordered or disordered could not be answered.

Taking into account that pressure has been shown to influence hydrogen bonding largely, in both solid^11,19–21^ and fluid^22^ phases, it is therefore highly desirable to study the structure of at least one alkali hydroxide hydrate as a function of pressure, with particular interest in the H-bond dynamics and the configurations adopted by the H-bond network as a response to increased density as well as the ‘guest’, ionic species. Such an endeavour would complete the picture provided thus far by studies of clathrate hydrates, where the non-water species in the system are mainly non-polar, such as Ne, H_2_,^23^ CH_4_,^24^ CO_2_,^25^ and other light-gas molecules.

In the case of hydrates, there is always the question of their thermodynamic stability field, with important consequences on the possible structures they may adopt and their exact chemical composition, which influences their physical and chemical properties. There is a general expectation that at a combination of pressure and temperature, all hydrates and molecular solids would exhibit dissociation, causing the compound to separate into its pure constituents. This behaviour has been well studied in the case of gas hydrates^26^ and pure nitrogen,^27^ but currently there is no equivalent information for alkali-hydroxide hydrates.

## 1 Methods

### 1.1 Sample preparation for X-ray and Raman experiments

Powder samples of CsOH·H_2_O (99.95%; Sigma-Aldrich, CAS Number 35103-79-8) were loaded in a clamp-type diamond anvil cell. For XRD experiments, type-Ia natural diamonds with a 0.6 mm culet (conical-shaped Böhler-Almax type) were used with a stainless steel (SUS301) plate as a gasket after preindentation from an initial thickness of 0.2 mm to 0.11 mm. A hole of 0.3 mm in diameter was mechanically drilled for the sample space. For Raman experiments, type-IIa diamonds with a 0.8 mm culet were used with a stainless steel (SUS304) plate after preindentation from an initial thickness of 0.3 mm. A hole of 0.4 mm in diameter was laser-drilled. Compression and decompression were performed by manual (un)tightening of screws. The sample pressure was determined by the ruby-fluorescence method.^28,29^

### 1.2 X-Ray diffraction (XRD)

X-ray diffraction patterns were measured with the micro-focussed X-ray source with a rotating Mo target (Rigaku: MicroMax007), a monochromator for the *Kα* line, confocal mirror (Rigaku), and a 0.05 mm single pinhole. The diffraction patterns were collected with imaging plates (Rigaku; *R*-axisIV^++^). The collection time was 10 min per data point, allowing for a ±10 degree horizontal oscillation.

### 1.3 Neutron diffraction (ND)

First, an ambient-pressure and temperature measurement was performed to check the required collection times, the quality of data (given the sample was hydrogenous), and to determine phase purity. 6 g of CsOH·H_2_O powder was loaded into a vanadium can in the glove box at the PLANET beamline of the J-PARC MLF.^30,31^ A 6-hour collection proved sufficient for these purposes and a single-phase pattern of caesium hydroxide monohydrate of sufficient quality for structure refinement was obtained (Fig. 2).

**Fig. 2:**
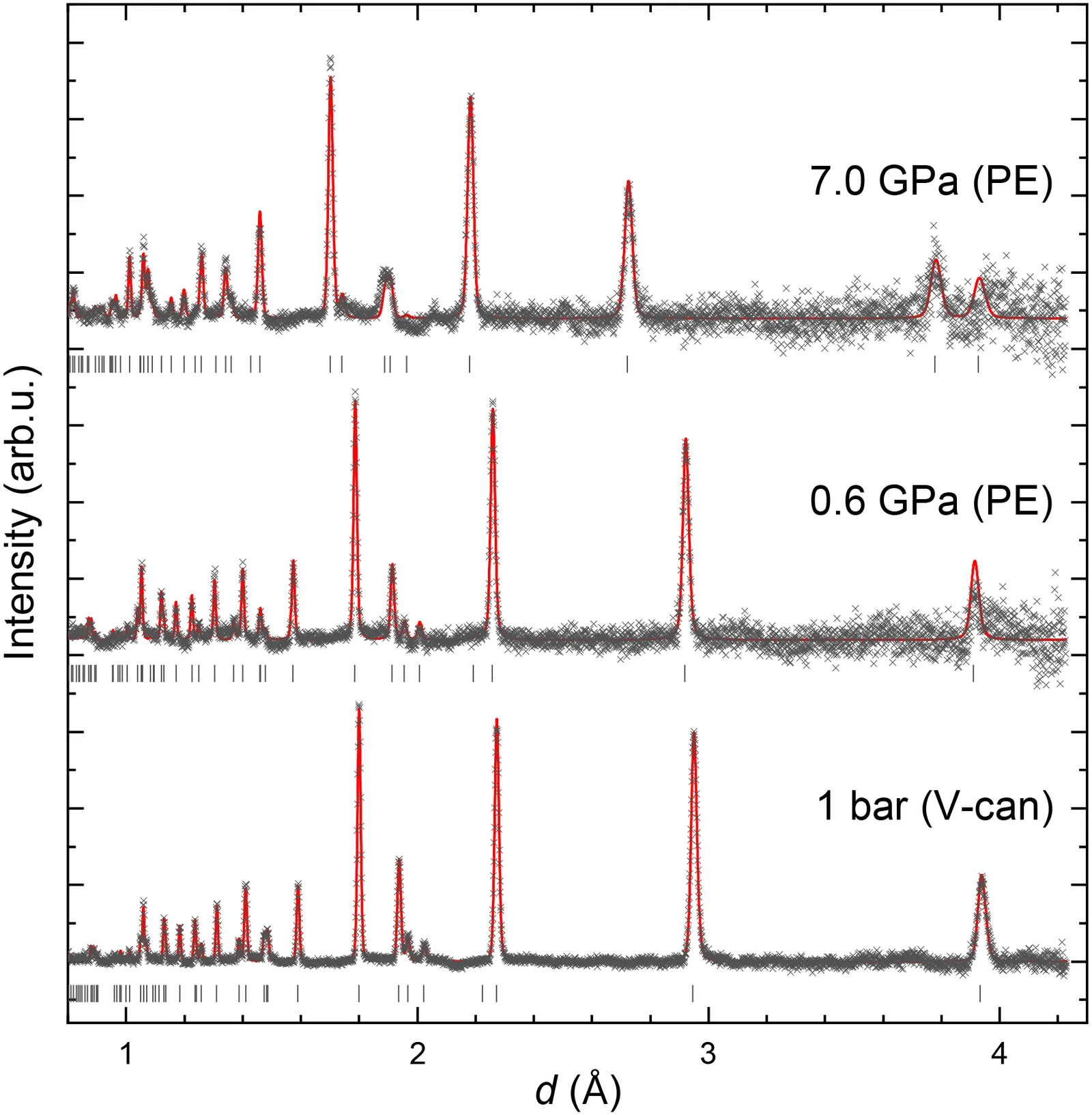
Representative neutron diffraction profiles at ambient conditions in a vanadium can and under pressure at 0.6 and 7.0 GPa in the PE press. Cross symbols, red lines, and ticks correspond to the observed intensities, calculated intensities from the Rietveld analysis with the *P*3*m*1 model, and calculated peak positions, respectively. The background curve derived in the Rietveld refinement is subtracted from the profiles for clarity.

Next, a pellet of CsOH·H_2_O was made in the glove box for high-pressure measurement. The pellet was loaded into a Paris–Edinburgh (PE) press with tungsten carbide (WC) anvils. The anvils were clamped with a force of 2 tonnes to seal the sample. The anvil assembly was put into the PE press, which was placed on the beamline. Diffraction patterns were collected for 12 hours each. The load was increased in 10-tonne steps up to a maximum of 60 tonnes. All of these measurements were performed at ambient temperature, and no decline in data quality was noticed. The sample pressures are estimated from the unit-cell volume of the sample based on the equation of state derived from the XRD data.

### 1.4 Density functional theory (DFT) calculations

The compression behaviour of CsOH·H_2_O was also examined by static-structure optimisation using CASTEP 25.11^32^ at a range of pressures. We used rSCAN functionals^33^ with on-the-fly norm-conserving pseudopotentials. Because of the requirement for a proton-ordered structure in the DFT calculation, we used a *Cm* model (Fig. 1), which is the only possible ordered configuration within the primitive cell: larger supercells give similar energies, as anticipated from the observed disordering.^34^

This *Cm* structure fits well with the low-temperature structure of CsOH·H_2_O claimed from the XRD study,^14^ aside from small symmetry-breaking easily removed by disorder. Since the symmetry difference gives additional anisotropy in plane, we mainly focus on the overall change in structure, such as in-plane and out-of-plane compression behaviours.

### 1.5 Raman scattering

Raman scattering was measured using a micro-Raman system. A 514.5 nm argon ion laser (Coherent Innova 70C) was used for excitation. The laser beam is focussed onto the sample to a few microns in diameter on a confocal optical microscope. The spectrometer is equipped with a spectrograph (Princeton Instruments; Isoplane SCT320) and cryogenically cooled CCD camera (Princeton Instruments; PyLoN 100). Each final spectrum was obtained by averaging three spectra with 30 or 60 s exposures for OH-stretching and low-frequency regions, respectively.

## 2 Results and discussions

### 2.1 X-ray and neutron diffraction

The equation of state was obtained from X-ray diffraction measurements during a cycle of compression and decompression. Upon compression up to 10 GPa, CsOH·H_2_O does not show any obvious feature of a phase transition. Since the absence of a pressure-transmitting medium may lead to non-hydrostatic conditions inside the sample chamber, the lattice parameters may deviate from the ideal ones. The anisotropic compression behaviour was in agreement with the decompression behaviour in the X-ray data (Fig. 3).

**Fig. 3:**
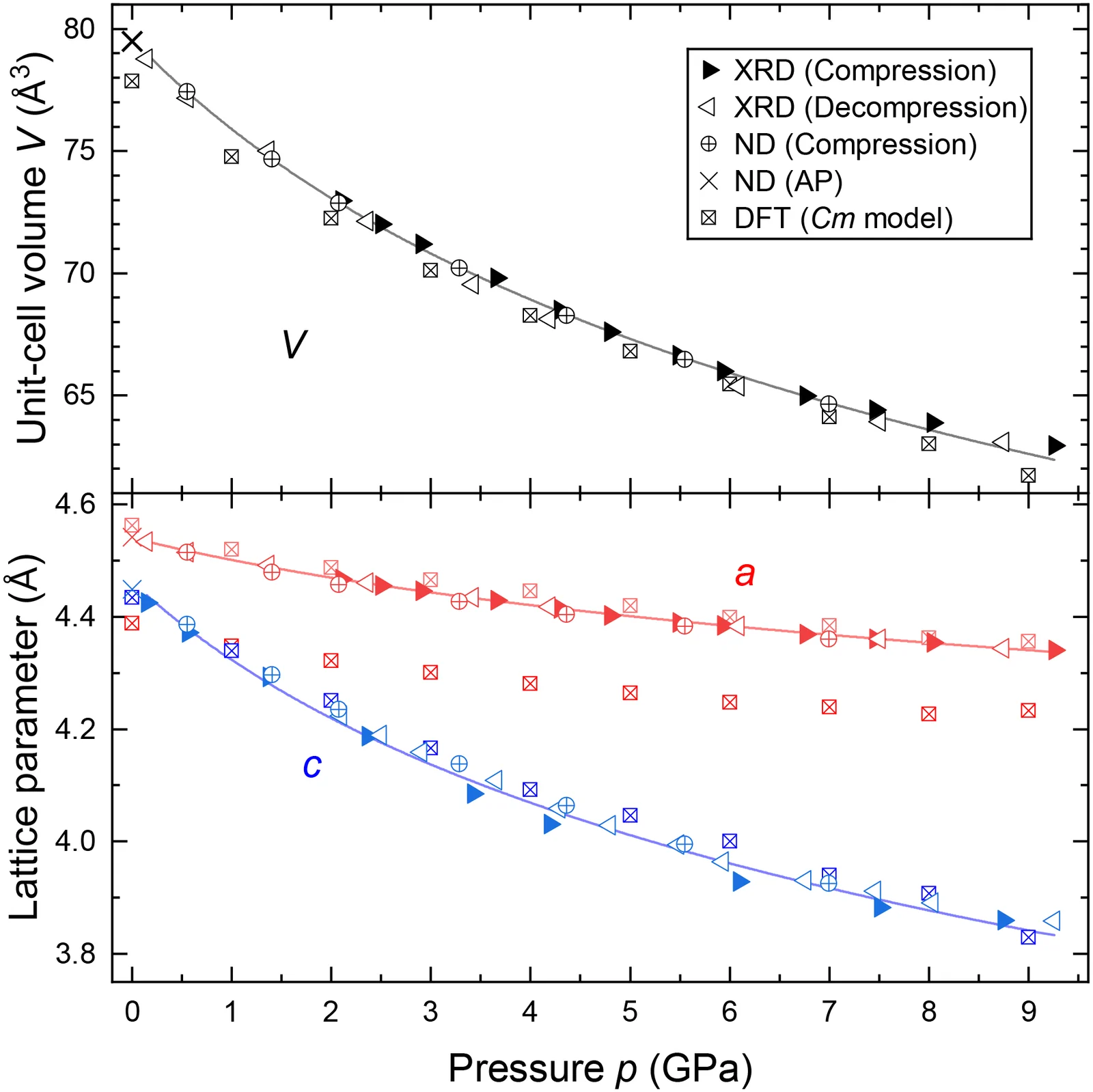
Lattice parameters at ambient temperature. MILEOS fitting curves (solid lines) are derived from XRD data for both compression and decompression. Pressures for neutron data were derived based on the volume EoS determined by the XRD data. Since the DFT optimisation is not constrained to have trigonal symmetry, the unit axes in the *a*–*b* plane are inequivalent, giving two distinct lattice parameters corresponding to *a* in the trigonal unit cell.

No clear evidence of a phase transition, such as the emergence/disappearance of peaks, was observed in neutron diffraction (Fig. 2), despite it probing the structural change in the H-sublattice like water ice.^15^ The previous neutron study on this hydrate^16^ indicated that the *P*3*m*1 model is more suitable than the *P*3̄*m*1 model. Our ambient-condition measurement also showed smaller *R*-factors in the Rietveld refinement, *R*_wp_ = 0.60% and *χ*^2^ = 1.53 with the *P*3*m*1 model against the *R*_wp_ = 0.66% and *χ*^2^ = 1.85 with the *P*3̄*m*1 model. This non-equivalent site model, distinguishing the hydroxyl ion and water molecules by hydrogen site occupancies of 1/3 and 2/3, also showed a larger oxygen displacement from the three-fold axis for the hydroxyl ion, as reported.^16^ Such site displacement from the average position is caused by the disordered molecular orientations, also seen in other molecular crystals such as ice VII.^35,36^ Nevertheless, the hydrogen site occupancies could not be determined freely, so in the *P*3*m*1 structural model, the hydrogen sites for H_2_O and OH^−^ were constrained to be symmetric, except for the fixed occupancies of 2/3 and 1/3. These constraints ensure a stable Rietveld refinement despite the high incoherent neutron scattering background.

Below we present the obtained equation of state for CsOH·H_2_O (Fig. 3). Since both the samples used for neutron and X-ray diffraction were hydrogeneous, the neutron results are shown on the same plot for comparison of anisotropic compression. There is a clear agreement between the results obtained with the two different techniques. The lattice parameter *a* shows a smaller change than the parameter *c*. This means that compression takes place mainly perpendicular to the hydrogen-bonded network.

To understand this anisotropic compression quantitatively, we derived the pressure dependence of the lattice parameters and volume by parameterising them using a Murnaghan integrated linear equation-of-state (MILEOS).^37^1
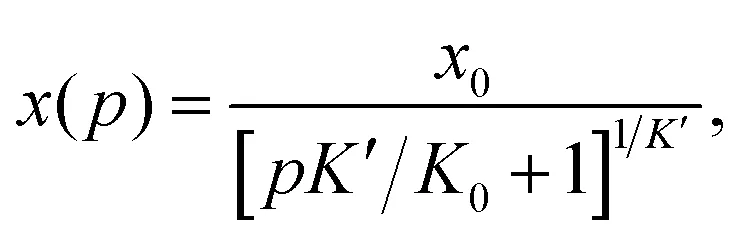
where *K*_0_ is the isothermal bulk modulus at 0 GPa and *K*′ is the first pressure derivative of the bulk modulus (∂*K*/∂*p*)_*p*=0_. The fitted parameters are summarised in Table 1. The lattice parameters extrapolated to 0 GPa show good agreement of lattice parameters with the ambient-pressure neutron data.

**Table 1:** Fitted parameters of the Murnaghan integrated linear equation of state (MILEOS; eqn (1))^37^ for CsOH·H_2_O. Pressures for neutron data were determined from the MILEOS fit of XRD data for both compression and decompression

			*x*_0_ a	*K*_0_ (GPa)	*K*′
XRD	All	*a* (Å)	4.5405 (16)	102 (4)	27.2 (11)
		*c* (Å)	4.462 (17)	27 (3)	9.6 (11)
		*V* (Å^3^)	79.6 (3)	18.4 (13)	5.3 (4)
XRD	Compression	*a* (Å)	4.551 (8)	82 (12)	32 (3)
		*c* (Å)	4.56 (6)	17 (5)	11.9 (12)
		*V* (Å^3^)	81.4 (10)	13 (3)	6.7 (6)
XRD	Decompression	*a* (Å)	4.5393 (18)	108 (5)	25.1 (18)
		*c* (Å)	4.465 (2)	26 (4)	9.4 (15)
		*V* (Å^3^)	79.6 (4)	18 (2)	5.2 (7)
ND	Compression	*a* (Å)	4.5434 (11)	77 (2)	34.9 (10)
		*c* (Å)	4.4545 (19)	33.9 (6)	7.1 (2)

a The neutron diffraction at ambient condition yielded lattice parameters of *a*_0_ = 4.54200 (5) Å, *c*_0_ = 4.44877 (13) Å, and *V*_0_ = 79.481 (2) Å^3^.

The MILEOS fitting highlights the anisotropy of the compression behaviour. The *K*_0_ value for the *a*-axis is several times larger than that along the *c*-axis. The large axial incompressibility of *K*_0_ in the order of 100 GPa is also found in some salt hydrates such as MgSO_4_·11H_2_O^38^ and MgCl_2_·7H_2_O.^39^ The highest compressibility is perpendicular to the layered structure in a similar manner to those in other layer-structured hydrous structures, such as graphite^40^ and brucite [Mg(OH)_2_].^41,42^ Interestingly, the pressure-derivative *K*′ along the *a*-axis is more than twice as large as that along the *c*-axis. This indicates that within the pressure regime below 10 GPa, the CsOH·H_2_O becomes even more incompressible in the *a*–*b* plane and compression occurs more and more anisotropically. To relieve this anisotropy, another trend change in phase transformation, such like the tetragonal structure^18^ or decomposition into ice and anhydrous salt at higher pressures, would take place at higher pressures.

Fig. 4 summarises the pressure dependence of local geometries based on the *P*3*m*1 model. By compression up to 7.0 GPa, Cs–O distances only decrease slightly from 3.22 Å to 3.11 Å (−3.6%). MILEOS parametrisation of the Cs–O distance gives the value of *K*_0_ ≈ 93 GPa for neutron data. This value is similar to the derived parameter for the *a*-axis (Table 1), implying the in-plane compression behaviour of CsOH·H_2_O is dominated by the nature of the Cs–O sheet structure.

**Fig. 4:**
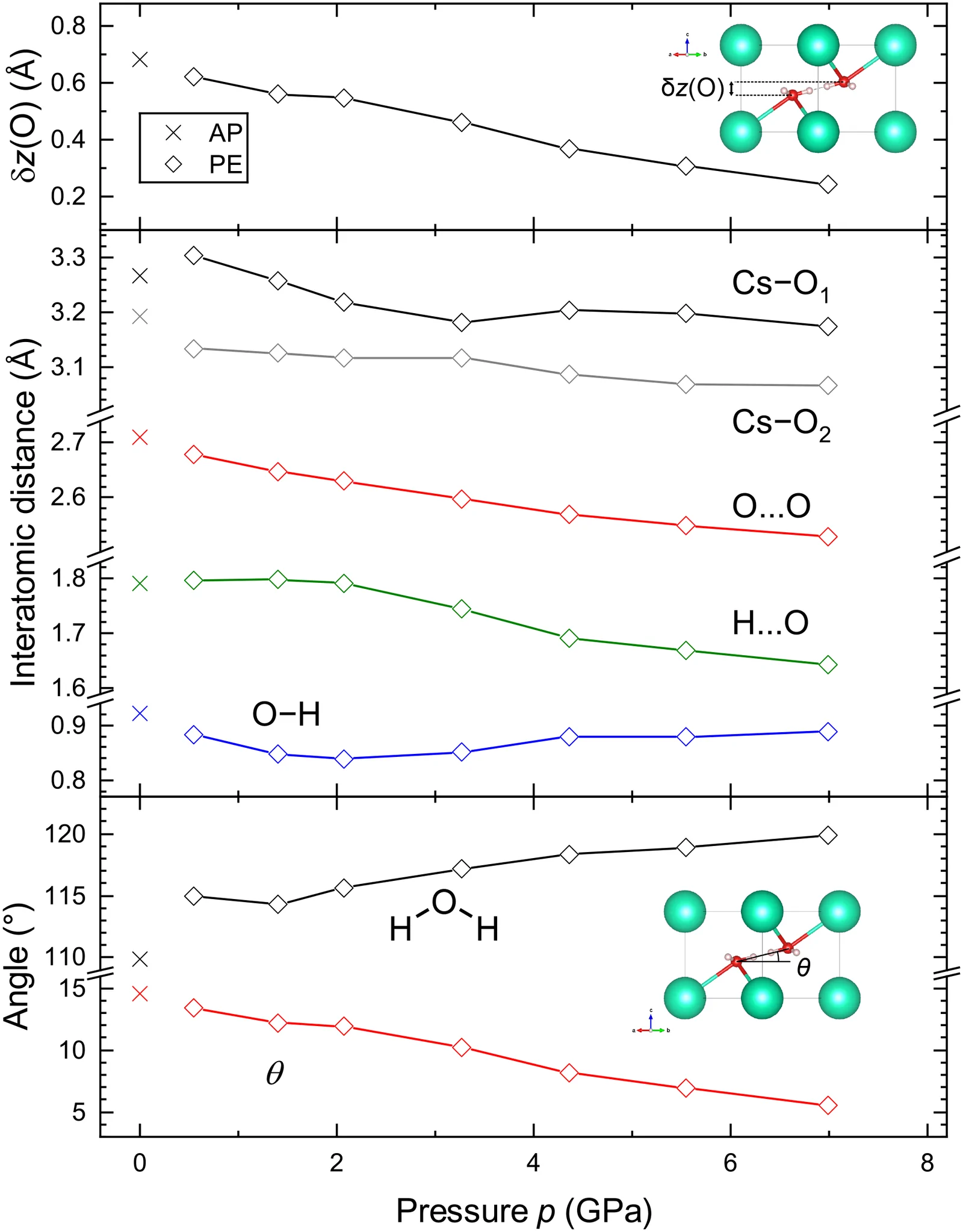
Geometric parameters from the Rietveld refinements derived from the neutron data using the *P*3*m*1 model. O_1_ and O_2_ correspond to the oxygen of H_2_O and OH^−^, respectively.

At ambient conditions, the pseudo-2D hexagonal network layer formed by water molecules and hydroxyl ions is not completely flat. Instead, the layer is buckled, represented as a positional difference between the neighbouring oxygens along the *c*-axis (δ*z* (O) = 0.16 Å). Hydrogen is located between the oxygens to mediate the hydrogen bonds. The three-fold hydrogen sites and the central oxygen site on the 3-fold axis form very flat triangular pyramids. With increasing pressure, the oxygen moves to the middle of the layer, and the hydrogen atoms move outwards from the 3-fold axis in the basal plane. This flattening of the hydrogen-bonded network is represented by the decrease of δ*z* (O) and the elevation angle of the neighbouring O⋯O from the *a*–*b* plane (*θ*) as well as the apparent intramolecular H–O–H angle reaching 360°/3 = 120°.

The comparison of the axial compressibility and pressure-dependence of local geometry and the overall compression behaviour of CsOH·H_2_O can be interpreted as the combination of the rigid layer structures of Cs–O bridges and hydrogen bonds in the *a*–*b* plane and softer inter-layer interactions along the *c*-axis. The hydrogen and oxygen move in a direction almost perpendicular to the hydrogen bonds with increasing pressure. This deformation flattens the hydrogen-bonded network, which makes the structure stiffer against the in-plane deformation accompanying the direct repulsion of the straight O–H⋯O.

The apparent O–H distance in CsOH·H_2_O from the neutron diffraction is significantly shorter than expected for a water molecule^20,35,43^ or a hydroxide ion.^11^ This difference comes from the fact that the three hydrogen sites and the oxygen site have to accommodate orientational disorder. In our analysis, the oxygen atom, likely multi-site disordered around the 3-fold axis, is located at the average 
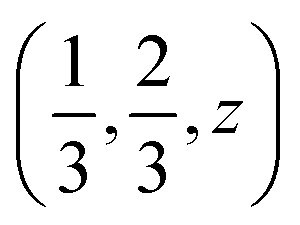
 site on the 3-fold axis. This results in the apparently short O–H distance as known for hydrogen-disordered ice phases.^35,36,43^ Thus, the pressure dependence of the apparent O–H distance likely reflects changes in the magnitude and/or direction of displacement of multi-site disorder rather than changes in the real covalent O–H bond length. It is worth mentioning that the atomic displacement parameters, especially of hydrogen sites, decrease upon compression (Table S2), implying changes in the atomic distribution in the disordered structure.

Adopting the deviation of the absolute value of O–H distances, their trend against compression should still be valid. The apparent O–H covalent bond distance shows little pressure effect, but may elongate above 2 GPa (Fig. 4). Other studies on true O–D bond lengths in systems with ordered sites have shown very small but positive pressure dependences for both hydroxide^11^ and water molecules.^20^ On the other hand, the hydrogen-bonded H⋯O distance is less sensitive up to 2 GPa but decreases at higher pressures. This trend change implies a shift from a low-pressure region dominated by geometrical distortion with flattening of the hydrogen-bonded network to a high-pressure region including simpler decreases in interatomic distances.

### 2.2 Raman scattering

Fig. 5 summarises the Raman profiles obtained on compression from 0.49 GPa to 3.73 GPa and decompression to 0.14 GPa at room temperature (296 K). At 0.49 GPa, the spectra reveal a sharp band at 3452 cm^−1^ due to the stretching vibration of hydroxide ions, as typical of hydroxide hydrates.^44,45^ Stretching modes of water molecules exist in the range 2400–3000 cm^−1^ in infrared absorption spectroscopy,^14,46^ but this is very weak and not visible in Raman scattering even at ambient conditions.^44^ In high-pressure measurements with a DAC, this region overlaps with the diamond Raman. In the low-frequency region below 1000 cm^−1^, several clear bands were observed at ≈50, ≈100, and 344 cm^−1^. The lower-frequency bands at ≈50 and ≈100 cm^−1^ are decomposed into several Lorentzian peaks as shown in Fig. 5. In addition to these strong bands, weak features are also observed as shallow bumps at ≈200, ≈670, ≈770, ≈810, ≈980 cm^−1^. According to the literature,^44,45^ the peaks below and above 400 cm^−1^ are assigned to translational and librational modes, respectively. However, some features do not perfectly match with the reported spectra,^44,45^ such as the bands at 40–50 cm^−1^, which were outside the measured frequency range.^44^ Instead of two tiny peaks at 148 and 159 cm^−1^,^44^ a weak feature was observed at ≈130 and ≈200 cm^−1^.

**Fig. 5:**
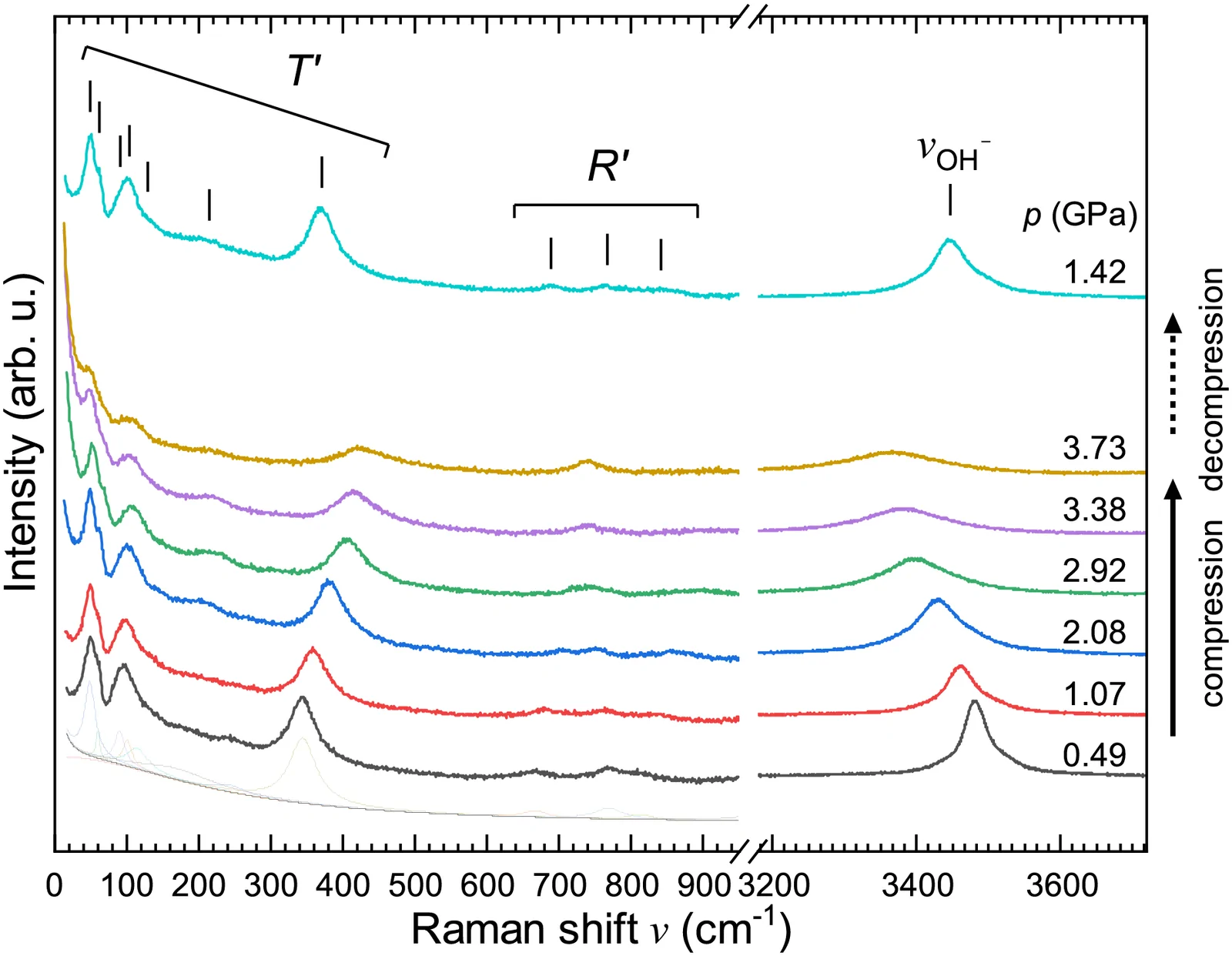
Representative Raman profiles upon isothermal compression at ambient temperature (296 K). *T*′, *R*′, and *ν*_OH^−^_ indicate the peak assignments of translation, libration, and OH stretching mode of the hydroxyl ion, respectively, based on the literature.^44^ The weak streching mode of H_2_O at 2400–3000 cm^−1^ is not visible hidden by the diamond Raman band. Tick marks indicate the peak positions assigned for the decompressed data. An example of fitted curves for the lower-frequency bands at the lowest pressure is shown at the bottom.

On compression, the OH stretching band shifts toward lower frequency (Fig. 5), which implies that hydrogen bonds become stronger by reducing the H⋯O distances^47^ in agreement with the neutron data. Such hydrogen-bond strengthening is also found in various hydrogen-bonded systems such as water ice^48^ and hydrous minerals.^49^ To characterise its response to compression, the peak positions are plotted in Fig. 6. The elastic properties can be characterised using the mode Grüneisen parameter (*γ*_*i*_). This parameter is determined from the volume (*V*) dependence of a given vibrational frequency. The definition for the *i*-th mode with Raman shift Δ*ν*_*i*_ is as follows:2
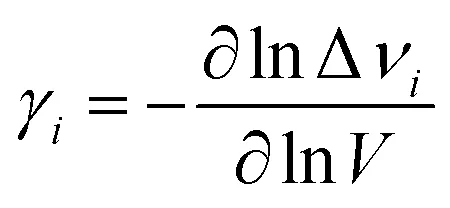


**Fig. 6:**
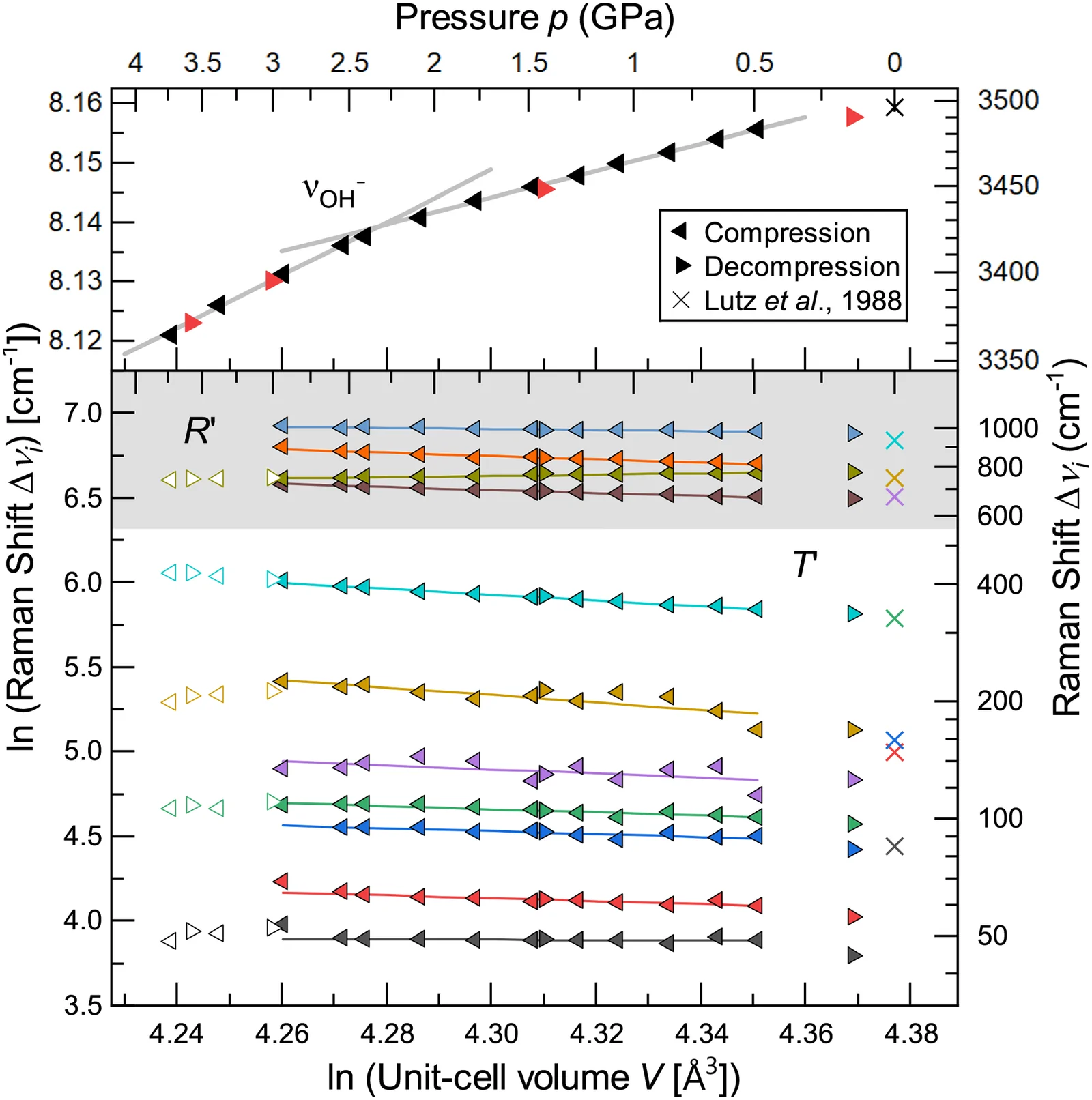
Grüneisen plot for the Raman bands corresponding to translation (*T*′), libration (*R*′), and OH stretch (*ν*_OH^−^_) modes observed at room temperature (296 K). The corresponding peak position and the pressure are described on the right and top axes, respectively. Grüneisen parameters are derived by fitting each band upon compression using eqn (2). The fits are shown in the solid lines. Ambient-pressure data^44^ are also plotted for comparison.

The volume is estimated from the equation of state derived from the XRD data (Table 1). Fig. 6 shows a clear linear relation of ln *V* − ln Δ*ν*_*i*_ over the whole pressure region of this study, although a kink appears at 2.3 GPa, where the decrease of the intermolecular H⋯O distance starts to level off (Fig. 4). Given that the softening of OH stretch mode occurs by the enhancement of hydrogen bonds,^44^ this can be connected to the pressure/volume dependence of geometry. Adopting the ambiguity from the disorder, the increase of OH covalent bonds and decrease in hydrogen-bonded H⋯O distance are dominant above 2 GPa (Fig. 4), which explain the larger effectivity of hydrogen-bond strengthening upon compression, connected to larger the Grüneisem parameter. The higher-pressure region gives the mode Grüneisen parameter of *γ*_OH,>2.3GPa_ = −0.444(16), almost doubled from *γ*_OH,<2.3GPa_ = −0.226(2) in the low-pressure region (Fig. 6 and Table 2). This change may reflect a change in the mechanism of compression of the hydrogen bonds. The mode Grüneisen parameter above 2.3 GPa is comparable to those for ice phases (*e.g.*, *γ*_OH_ = −0.36 for ice I_h_^50^ and *γ*_OH_ = −0.39 for ice VII^48^), crystalline phases which purely consist of hydrogen bonds. This implies a similarity in the compression behaviour of the hydrogen-bonded structure.

**Table 2:** Vibrational modes, their wavenumbers extrapolated to ambient pressure 
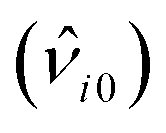
, pressure derivatives of the frequency of mode *i* (d*ν*_*i*_/d*p*)_*p*=0_, mode Grüneisen parameters (*γ*_*i*_), and corresponding assignments. Errors are derived from the standard deviation of the linear regression

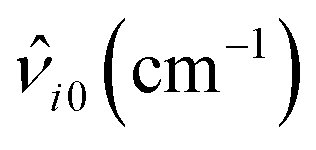	(d*ν*_*i*_/d*p*)_*p*=0_ (cm^−1^ per GPa)	*γ*_*i*_	Assignment
47.5 (9)	1.1 (5)	0.10 (13)	Translation
57.9 (8)	2.9 (5)	0.88 (13)	Translation
87.6 (8)	3.0 (5)	0.84 (16)	Translation
99.5 (10)	3.5.6 (5)	0.90 (13)	Translation
124 (5)	5 (3)	1.2 (6)	Translation
179 (6)	16 (3)	2.2 (5)	Translation
332.8 (8)	24.4 (5)	1.75 (5)	Translation
656.4 (15)	22.8 (8)	0.88 (3)	Libration
774.9 (8)	–10.5 (5)	–0.375 (16)	Libration
796 (3)	31.4 (18)	0.98 (7)	Libration
977 (2)	12.5 (12)	0.33 (4)	Libration
3497.5 (9)	–32.2 (7)	–0.226 (2)	OH stretch of OH^−^ (<2.3 GPa)
3525 (4)	–42.9 (12)	–0.444 (16)	OH stretch of OH^−^ (>2.3 GPa)

Regarding the translation and libration modes in the low-frequency region, the profile features remain almost intact up to 3 GPa, but the peaks become weak and blurred above 3 GPa (Fig. 5). Especially, the librational band at 650–700 cm^−1^ become unrecognisable and the shoulder at 60–70 and 110–130 cm^−1^ are resolveable. Upon decompression to below 3 GPa, the Raman features are recovered. This pressure is slightly higher than where the kink is observed for the OH bands with pressure (Fig. 6). Whilst this frequency reduction may imply some changes in the hydrogen-bonding structure or its dynamic behaviour, it should be noted that we cannot exclude the possibility of nonhydrostatic stresses in the sample chamber.

Below 3 GPa, the lattice and librational modes shift to higher frequency, except for a mode at ≈770 cm^−1^. Among the translational modes, the bands above 100 cm^−1^ show relatively larger mode Grüneisen parameters (Table 2), and libration modes show comparable or smaller mode Grüneisen parameters compared to the translational modes. The difference in the mode Grüneisen parameters provides geometric information about the bands. For example, graphite^40^ has two Raman-active modes, one at 42 cm^−1^ for interlayer shear modes and the other at 1581 cm^−1^ containing in-plane displacement. The graphite structure is also compressed mainly by decreasing the interlayer distance, which results in a large mode Grüneisen parameter of 3.7, in contrast to the close-to-zero mode Grüneisen parameter of 0.1 for in-plane displacement. The structure of CsOH·H_2_O is slightly more complicated since the structure is not the stacking of perfect 2D-layers but consists of the finite-sized Cs–O layers and inter-layer hydrogen bonds.

As with other layered structures with interlayer hydrogen bonds, the values of *γ*_*i*_ ≈ 1 for the translational modes observed for CsOH·H_2_O are similar to those of brucite (Mg(OH)_2_) and portlandite (Ca(OH)_2_),^49^ which have MO_6_ (M: cations) layers and interlayer hydrogen bonds. The mode Grüneisen parameters of CsOH·H_2_O are slightly larger than brucite (0.65–0.81). This is consistent with the more compressible nature of CsOH·H_2_O compared to brucite,^41^ but similar manner to portlandite,^49,51^ (isostructure, but softer than brucite). In the case of brucite, the translation modes corresponding to the in-plane vibrations of the O–H units exhibit larger Grüneisen parameters than those for out-of-plane vibrations. In a similar manner, translation modes with larger mode Grüneisen parameters contain an in-plane movement component.

## 3 Conclusions

We examined the pressure effect on the structure and dynamics of CsOH·H_2_O *via* diffraction and Raman spectroscopy as well as DFT calculations. CsOH·H_2_O shows anisotropic compression, with the most compressible axis being *c*. This leads to a significant decrease in the interlayer distance by 13% at 9 GPa, compared with the smaller decrease of in-plane distances (4.4%), similar to other layered structures.^40–42^ Under pressure, the buckled H_2_O–HO^−^ layer becomes flatter, tending towards a 2D hexagonal layer. The OH stretch Raman frequency downshifts on compression, implying the strengthening of hydrogen bonds. On the other hand, other vibration frequencies upshift on compression.

It was already established that, at ambient pressure, CsOH·H_2_O exhibits a series of temperature-driven phase transitions.^14,16,44^ In addition to rapid proton dynamics, these transitions involve increasing disorder of the OH and H_2_O molecules, and irregularity in the buckling.^34,52^ By contrast, we show here that compression reduces movement of hydrogen. The geometrical change of the quasi-2D hydrogen-bonded network into a flatter structure will give another insight into the structural relation between the free volume in the lattice and the dynamic behaviour of hydrogen, in addition to the relation of one-to-one relation to interatomic distance mostly focussed on in the ice studies to date.^53^

## Author contributions

C. G. Pruteanu: conceptualization, data curation, formal analysis, funding acquisition, investigation, methodology, project administration, validation, visualization, writing – original draft. K. Yamashita: data curation, formal analysis, investigation, methodology, project administration, validation, visualization, writing – original draft. G. J. Ackland: data curation, formal analysis, methodology, resources. K. Komatsu: data curation, formal analysis, funding acquisition, investigation, methodology, resources. T. Hattori and S.Machida: investigation, resources. J. S. Loveday: conceptualization, funding acquisition, project administration, resources, supervision, validation. All authors: writing – review & editing.

## Conflicts of interest

There are no conflicts to declare.

## Supplementary Material

CP-OLF-D6CP03154D-s001

## Data Availability

The data supporting this article have been included as part of the supplementary information (SI). Supplementary information: lattice parameters obtained by the Rietveld analysis of the XRD and neutron diffraction, atomic coordinates from neutron diffraction, and Raman frequencies. See DOI: https://doi.org/10.1039/d6cp03154d.
