# Supplementary material for: The high-pressure behaviour of caesium hydroxide monohydrate
Source: Phys Chem Chem Phys. 2026 Sep 24. Online ahead of print. doi: 10.1039/d6cp03154d (PMC13701514; doi:10.1039/d6cp03154d)
Supplement: Supplementary file 1 [file CP-OLF-D6CP03154D-s001.pdf]

Supplementary Information (SI) for Physical Chemistry Chemical Physics.
This journal is © the Owner Societies 2026

Supporting Information for Publication

The high-pressure behaviour of caesium hydroxide monohydrate

Keishiro Yamashita,*[a] Ciprian G. Pruteanu,*[a] Graeme J. Ackland,[a] Kazuki Komatsu,[b] Takanori Hattori,[c] Shinichi Machida[d] and John S. Loveday[a]

[a] SUPA, School of Physics and Astronomy and Centre for Science at Extreme Conditions, James Clerk Maxwell Building, Peter Guthrie Tait Road, University of Edinburgh, Edinburgh EH9 3FD, UK.

[b] Geochemical Research Center, Graduate School of Science, The University of Tokyo, Bunkyo-ku, Tokyo 113-0033, Japan.

[c] J- PARC Center, Japan Atomic Energy Agency, 2-4 Shirakata, Tokai, Naka, Ibaraki 319-1195, Japan.

[d] Neutron Science and Technology Center, Comprehensive Research Organization for Science and Society (CROSS), IQBRC Building, 162-1 Shirakata, Tokai, Naka, Ibaraki 319-1106, Japan.

*e-mail: kyamashi@ed.ac.uk; cip.pruteanu@ed.ac.uk

S1 High-pressure x-ray and neutron diffraction

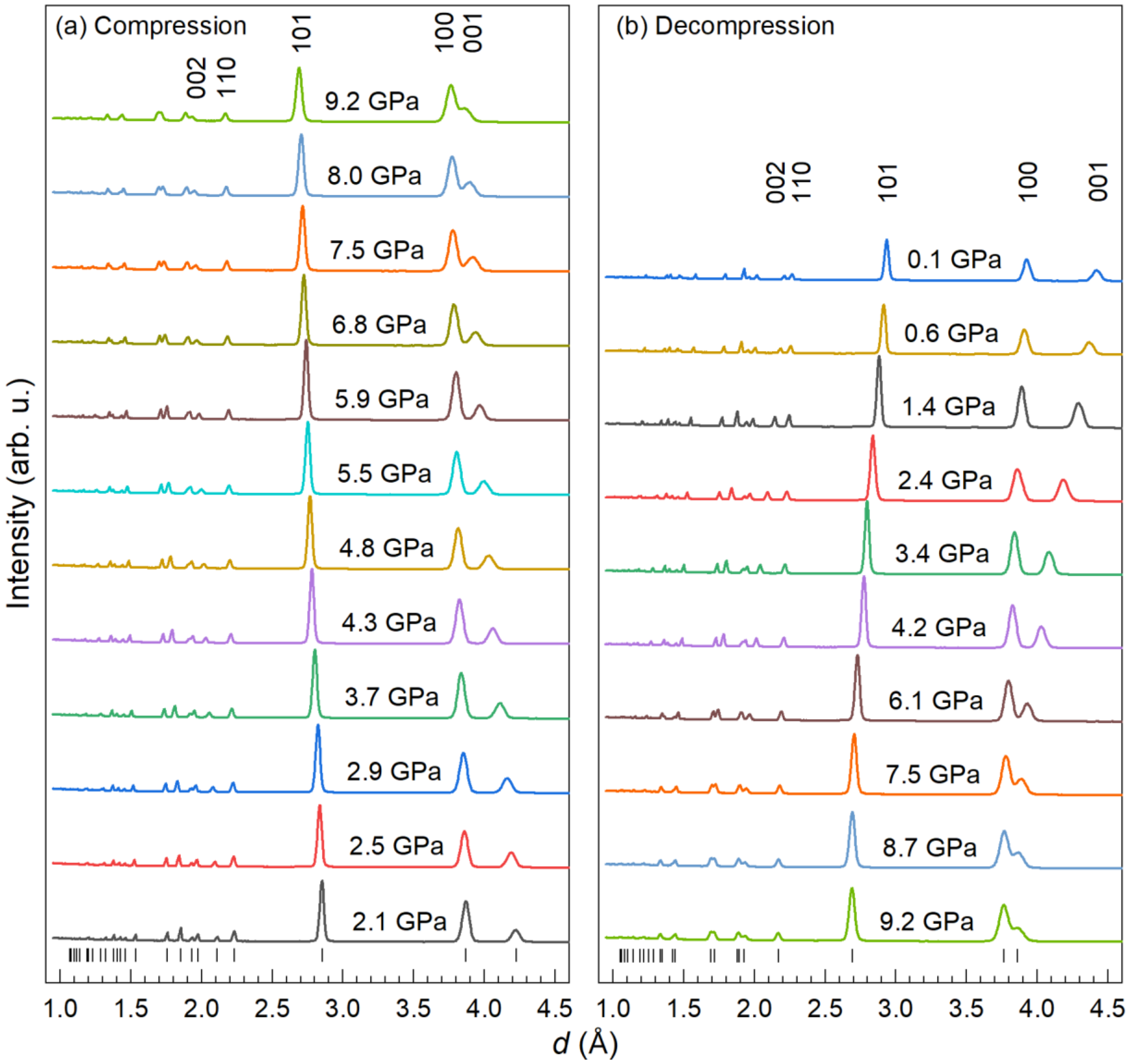

Figure S1 X-ray diffraction patterns upon (a) compression and (b) decompression. Calculated peak positions for $CsOH{\cdot}H_2O$ at (a) 2.1 and (b) 9.2 GPa are shown by ticks at the bottom. The anisotropic compression behaviour is seen as the drastic shifts of the 001 and 002 peaks compared to the 100 and 110 peaks. The pressure is estimated from the ruby fluorescence method.[1]

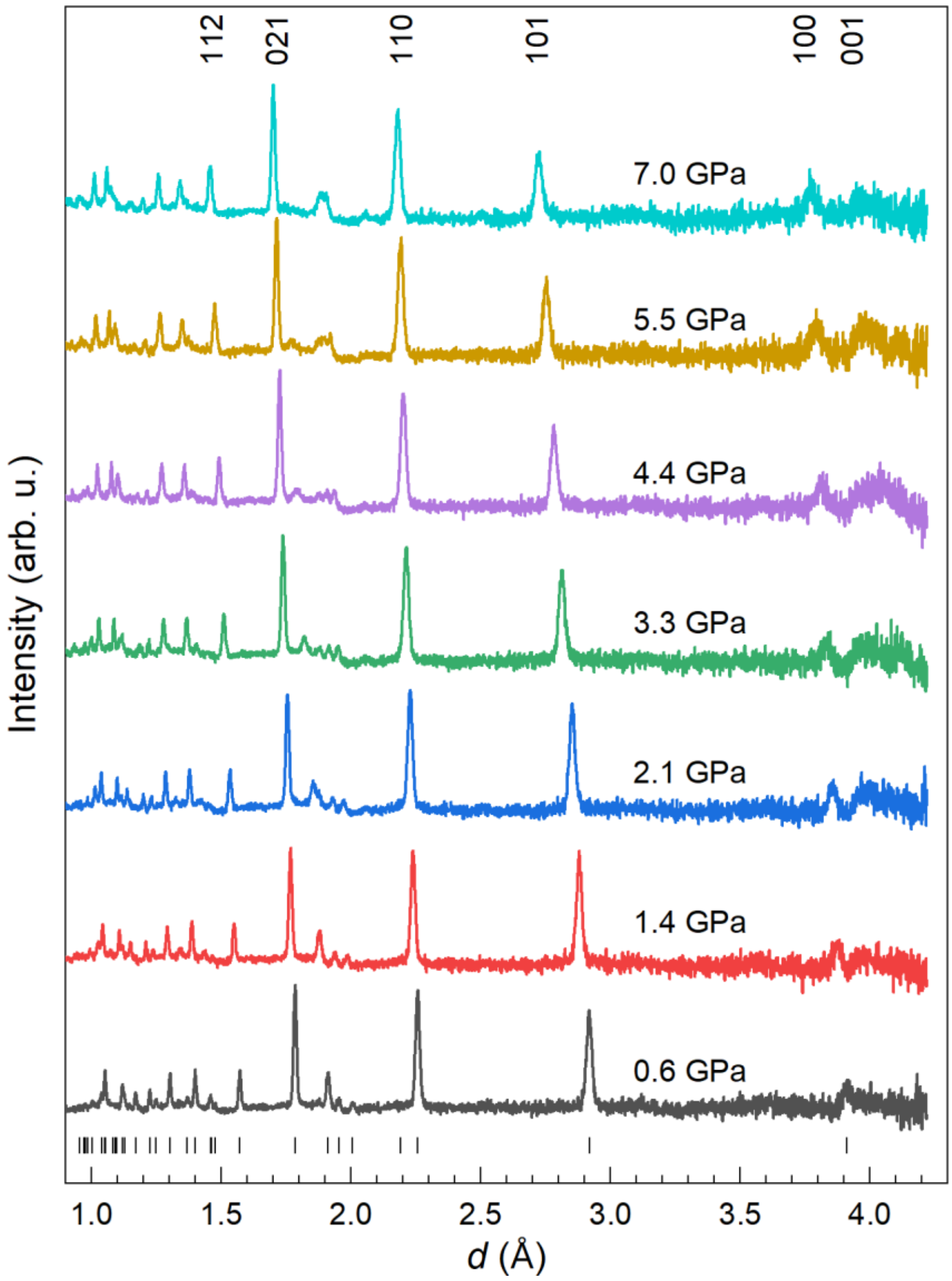

Figure S2 Neutron diffraction patterns upon compression. The background curve is subtracted for clarity. Calculated peak positions for $CsOH \cdot H_2O$ at the lowest pressure are shown by ticks at the bottom. Pressure is extimated from the volume Equation of State derived from the XRD data (Table 1)

Table S1 Lattice parameters derived by the Rietveld refinement for x-ray (XRD) and neutron (ND) diffraction using the structure model of *P*3*m*1.

	*p** (GPa)	*a* (Å)	*c* (Å)	*V* (Å^3)
XRD (Compression)	2.1	4.46628 (11)	4.22349 (16)	72.961(3)
	2.5	4.45476 (9)	4.18938 (12)	72.000(2)
	2.9	4.44550 (12)	4.15916 (15)	71.183(3)
	3.7	4.42891 (14)	4.10897 (18)	69.800(4)
	4.3	4.41401 (15)	4.05833 (19)	68.477(4)
	4.8	4.40181 (17)	4.02874 (21)	67.602(4)
	5.5	4.38956 (16)	3.99370 (21)	66.642(4)
	5.9	4.38458 (15)	3.96402 (18)	65.997(4)
	6.8	4.36910 (17)	3.93005 (21)	64.970(4)
	7.5	4.36038 (14)	3.91129 (17)	64.402(5)
	8.0	4.35369 (14)	3.89102 (17)	63.872(4)
	9.3	4.34062 (16)	3.85810 (20)	62.952(5)
XRD (Decompression)	8.7	4.34388 (15)	3.85989 (17)	63.076(5)
	7.5	4.36052 (14)	3.88270 (16)	63.935(4)
	6.1	4.38328 (11)	3.92883 (13)	65.372(3)
	4.2	4.41794 (11)	4.03077 (12)	68.133(3)
	3.4	4.43388 (11)	4.08442 (12)	69.539(3)
	2.4	4.46019 (12)	4.18691 (14)	72.132(4)
	1.4	4.49197 (8)	4.29214 (10)	75.003(2)
	0.6	4.51453 (12)	4.37194 (15)	77.167(3)
	0.1	4.53393 (13)	4.42509 (17)	78.777(4)
ND (Compression)	0.6	4.51473 (12)	4.3859 (3)	77.420(5)
	1.4	4.47956 (14)	4.2961 (3)	74.659(6)
	2.1	4.45721 (15)	4.2350 (4)	72.863(6)
	3.3	4.42664 (16)	4.1379 (4)	70.220(7)
	4.4	4.40422 (16)	4.0641 (4)	68.270(7)
	5.5	4.38323 (17)	3.9955 (5)	66.479(7)
	7.0	4.36043 (18)	3.9258 (5)	64.642(7)

*Pressures are derived from the ruby fluorescence method[1] for XRD and the volume equation of state from the XRD data (Table 1) for ND.

Table S2 Fractional coordinates derived by the Rietveld refinement for neutron diffraction using the structure model of *P*3*m*1.

p*	z_{Cs}	U_{Cs}	z_O†	U_{O1}	U_{O2}	x_H†	z_H†	U_H
0.6	0.033 (6)	0.011 (2)	0.5707 (10)	0.026 (3)	0.065(6)	0.4433 (6)	0.5246 (16)	0.0250 (13)
1.4	0.026 (8)	0.015 (3)	0.5652 (10)	0.020 (3)	0.052(6)	0.4393 (7)	0.5173 (18)	0.0306 (14)
2.1	0.020 (11)	0.017 (3)	0.5643 (10)	0.020 (4)	0.048(6)	0.4396 (6)	0.522 (2)	0.0294 (13)
3.3	0.013 (19)	0.016 (2)	0.5555 (11)	0.023 (4)	0.042(6)	0.4428 (6)	0.520 (2)	0.0179 (12)
4.4	0.024 (8)	0.010 (2)	0.5451 (15)	0.026 (4)	0.041(6)	0.4476 (6)	0.517 (2)	0.0099 (11)
5.5	0.027 (8)	0.013 (3)	0.5383 (19)	0.022 (4)	0.042(7)	0.4486 (6)	0.515 (3)	0.0058 (16)
7.0	0.023 (9)	0.012 (2)	0.531 (2)	0.015 (3)	0.032(5)	0.4510 (6)	0.524 (3)	0.0015 (11)

*Pressures are derived based on the volume equation of state from the XRD data (Table 1).

†The oxygen and hydrogen site positions are constrained to be symmetric between H_2O (H1 and O1 with occupancy of H1 = 2/3) and OH (H2 and O2 with occupancy of H2 = 1/3) in the *P*3*m*1 model as

$$z\ (\mathrm{O}_1) = 1 - z\ (\mathrm{O}_2) = z_{\mathrm{O}}$$
$$x\ (\mathrm{H}_1) = 1 - x\ (\mathrm{H}_2) = x_{\mathrm{H}}$$
$$z\ (\mathrm{H}_1) = 1 - z\ (\mathrm{H}_2) = z_{\mathrm{H}}$$

S2 High-pressure Raman scattering

Table S3 Raman frequencies of observed bands (cm^{-1}).

*p** (GPa)	*V*† ($Å^3$)	Translation							Libration				OH stretch
Compression													
0.49	77.54	48.6	59.7	89.8	100.4	114.6	168.8	344.3	667.0	769.4	812.8	984.1	3483.0
0.65	76.96	49.7	61.4	89.3	102.0	135.4	188.5	349.1	670.6	769.4	819.3	983.8	3477.2
0.85	76.24	47.7	60.0	91.6	103.8	133.4	204.4	353.1	675.6	766.3	823.7	989.2	3469.5
1.07	75.51	48.8	61.0	88.5	100.7	125.8	211.0	359.4	683.4	763.4	833.1	989.7	3462.8
1.26	74.93	48.8	61.4	90.6	103.4	136.0	200.2	364.5	684.3	760.7	834.8	992.1	3455.8
1.48	74.29	48.7	61.4	92.9	105.1	124.7	206.9	369.5	687.6	760.5	844.2	995.0	3449.0
1.77	73.48	48.5	62.3	92.5	106.8	140.3	201.7	375.4	696.6	754.2	842.8	997.3	3441.3
2.08	72.70	48.9	62.8	94.8	108.4	143.9	210.9	382.0	705.6	751.3	859.0	1006.6	3431.2
2.41	71.94	49.2	63.8	94.8	108.4	138.1	220.9	390.5	709.6	750.3	869.8	1008.3	3420.7
2.55	71.63	49.3	65.1	95.1	108.5	134.7	217.7	393.7	718.4	749.4	875.0	1001.6	3415.5
2.93	70.83	53.5	69.0	-	107.8	134.2	224.3	406.6	720.2	743.9	897.2	1017.5	3399.1
3.38	69.96	50.5	-	-	106.3	-	207.3	416.9	-	741.1	-	-	3381.2
3.73	69.33	48.5	-	-	106.3	-	198.2	427.1	-	739.8	-	-	3364.1
Decompression													
3.58	69.59	51.3	-	-	108.0	-	205.8	425.8	-	740.4	-	-	3370.9
3.01	70.67	52.2	-	-	110.5	-	211.4	409.9	-	746.0	-	-	3395.4
1.42	74.44	49.1	62.1	92.1	104.3	129.6	213.5	370.5	690.0	767.7	841.6	991.9	3448.1
0.14	78.97	44.4	55.8	83.0	96.5	125.5	168.1	333.6	658.2	774.1	-	968.9	3490.0

*Pressures are derived from the ruby fluorescence method.[2]

†Unit-cell volume is calculated based on the equation of state derived by the XRD.

References for Supplemental Information

1 G. J. Piermarini, S. Block, J. D. Barnett and R. A. Forman, Calibration of the pressure dependence of the R_1 ruby fluorescence line to 195 kbar, *J. Appl. Phys.*, 1975, **46**, 2774–2780.

2 G. Shen, Y. Wang, A. Dewaele, C. Wu, D. E. Fratanduono, J. Eggert, S. Klotz, K. F. Dziubek, P. Loubeyre, O. V. Fat'yanov, P. D. Asimow, T. Mashimo and R. M. M. Wentzcovitch, Toward an international practical pressure scale: A proposal for an IPPS ruby gauge (IPPS-Ruby2020), *High Press. Res.*, 2020, **40**, 299–314.
